# Safety of Combined Face and Necklift: Does Opening the Neck Increase Risk? A Retrospective Analysis of Hematoma Risk

**DOI:** 10.1093/asjof/ojag138.015

**Published:** 2026-07-24

**Authors:** Nusaiba Baker, Jad Abi-Rafeh, Brian Bassiri-Tehrani, Lori Plonski, Foad Nahai

**Affiliations:** University of California, San Francisco, San Francisco, CA, USA; McGill University Health Center, Montreal, Quebec, Canada; Pearlman Aesthetic Surgery, New York, New York, USA; Metroderm & Center for Plastic Surgery, Atlanta, GA, USA; Metroderm & Center for Plastic Surgery, Atlanta, GA, USA; Emory University, Atlanta, GA, USA

## Abstract

**Goals/Purpose:**

The demand for face and necklifts has been steadily increasing as cultural and social media trends evolve. The safety of performing combined face- and necklift surgery has been scrutinized over the years, and the literature has carefully queried safe practice to avoid complications. Performing both a face- and necklift, with neck undermining, in a single surgery creates a larger dead space than either procedure alone, and the literature reflects an increased risk of hematoma in concomitant procedures. Here, we present a single surgeon’s experience with the implementation of strict perioperative blood pressure control after combined face- and necklift

**Methods/Technique:**

We reviewed 502 consecutive facelifts performed between 2004 and 2018 by a single surgeon to examine outcomes associated with concomitant necklift, defined as any submental incision and advanced neck contouring techniques. Hematoma incidence was compared between the facelift-only group (Group A) and combined face- and necklift (Group B) cohort and further stratified by operative date (before vs. after 2015) to assess the effect of strict postoperative blood pressure control using targets of

**Results/Complications:**

A total of 263 patients underwent facelift alone (Group A) and 238 underwent combined faceand necklift (Group B). The average age was 61±7.4 years in Group A and 63±6.9 years in Group B (p<0.01). A significantly higher proportion of male patients underwent the combined procedure (13% vs. 3.4%, p<0.01), and the mean BMI was demonstrated to be higher in Group B (24±3.8 vs. 23±3.3, p<0.01). Notably, no male patients in either group developed hematoma.

The hematoma rate was 4.2% (11/263) following facelift alone, and 1.3% (3/238) after combined face- and necklift (p=0.06). This suggests that adding a submental incision and deep plane neck dissection does not increase the risk of hematoma.

When evaluating cohorts with different perioperative systolic blood pressure targets, in patients for whom a <140mmHg target was used, hematoma rates were again found to be lower in the combined face- and necklift group (1.8% in Group B vs. 4.2% in Group A; p = 0.24). In patients for whom a <120mmHg target was used, no hematomas (0%) occurred in patients undergoing combined face and necklift, compared with a hematoma rate of 4.3% in facelift-alone cases, demonstrating a favorable trend associated with improved blood pressure management.

Among the combined face and necklift cohort, 33 patients (13.9%) also underwent deep neck procedures, including digastric reduction and submandibular gland resection. In patients who underwent digastric or submandibular gland reduction (n = 33), hematoma was infrequent (2/33; 6%), with both events occurring prior to 2015. Though not statistically significant (p=0.16), this trend further supports the safety of deeper cervical maneuvers when performed under stringent hemodynamic control.

**Conclusion:**

In this large series, opening the neck—even with advanced contouring techniques—did not increase hematoma risk. Hematoma remained rare, and with appropriate surgical technique and strict perioperative blood pressure control, decisions about submental incision and neck dissection should not be limited by concerns of added hematoma risk.

